# A Single Session of SMR-Neurofeedback Training Improves Selective Attention Emerging from a Dynamic Structuring of Brain–Heart Interplay

**DOI:** 10.3390/brainsci12060794

**Published:** 2022-06-17

**Authors:** Pierre Bouny, Laurent M. Arsac, Yvan Pratviel, Alexis Boffet, Emma Touré Cuq, Veronique Deschodt-Arsac

**Affiliations:** 1 Laboratoire IMS, CNRS, UMR 5218, Université de Bordeaux, 33400 Talence, France; laurent.arsac@u-bordeaux.fr (L.M.A.); yvan.pratviel@u-bordeaux.fr (Y.P.); alexis.boffet@outlook.fr (A.B.); veronique.arsac@u-bordeaux.fr (V.D.-A.); 2 URGOTECH, 15 Avenue d’Iéna, 75116 Paris, France; emma.toure@gmail.com

**Keywords:** brain waves, complexity, entropy, HRV, multifractality, selective attention, SMR neurofeedback

## Abstract

Research on sensorimotor rhythms (SMR) based on neurofeedback (NFb) emphasizes improvements in selective attention associated with SMR amplification. However, the long-term training proposed in most studies posed the question of acceptability, which led to the evaluation of the potential of a single NFb session. Based on cognitive and autonomic controls interfering with attention processes, we hypothesized changes in selective attention after a single SMR-NFb session, along with changes in brain–heart interplay, which are reflected in the multifractality of heartbeat dynamics. Here, young healthy participants (n = 35, 20 females, 21 ± 3 years) were randomly assigned either to a control group (*Ctrl*) watching a movie or to a neurofeedback (*NFb*) group performing a single session of SMR-NFb. A headset with EEG electrodes (positioned on C3 and C4) connected to a smartphone app served to guide and to evaluate NFb training efficacy. A Stroop task was performed for 8 min by each group before and after the intervention (movie vs. SMR-NFb) while collecting heart rate variability and C4-EEG for 20 min. When compared to *Ctrl*, the *NFb* group exhibited better Stroop performance, especially when facing incongruent trials. The multifractality and NFb training efficacy were identified as strong predictors of the gain in global Stroop performance, while multifractality was the only predictor regarding incongruent trials. We conclude that a single session of SMR-NFb improves selective attention in healthy individuals through the specific reorganization of brain–heart interplay, which is reflected in multifractal heartbeat dynamics.

## 1. Introduction

Neurofeedback training (NFb) has developed in recent years as a mental practice in which one is guided to generate selective brainwave magnifications during specific sessions. NFb has proven beneficial effects in patients suffering from hyperactivity, brain diseases, or sleep disorders. In healthy people, NFb is believed to enhance cognitive functions and attention processes and helps improve behavior and well-being. A number of brain waves have been targeted during NFb, among which sensory-motor rhythms (SMR) (12–15 Hz) [1,2,3,4] have been associated with improved attentional processes [5,6,7], even in healthy subjects [8,9] who did not suffer from any brain disease. A drawback with such interventions is that people have been submitted to several sessions of SMR-NFb [8,9,10], which poses the question of acceptability. Interestingly, recent findings point to potential benefits of a single NFb session; as an example, Gadea et al. [11] showed improved psychological markers (anxiety, depression, and anger) and biological markers (EEG, and cortisol) of calmness and anxiety. By showing that both subjective (psychometric) and objective (biological) markers changed congruently after a single session of SMR-NFb training, these results rule out a nonspecific (placebo) effect of a single SMR-NFb session. They confirm that SMR activity, generally associated with internal inhibition, may have links with a calm feeling, at least in calm resting conditions in their studies, which could rely on the anxiety dimension suggested by Gadea et al. [11]. How this possible phenomenon is connected with benefits for selective attention deserves further investigation because brain and heart activities during calm conditions do not resemble the neural processes that arise during cognitive activity.

In a number of daily activities, some cognitive resources are devoted to orienting attention. This represents a critical function through which one detects errors and selects information to prioritize for goal achievement. Attention is a complex neural and psychological phenomenon that unfolds across many brain structures and mechanisms [10,12,13]. As a multidimensional construct, attention cannot be reduced to a single measure of cognitive performance [14] but rather emerges from a dynamic structuring of neural interactions. According to the concept of neurovisceral integration developed by Benarroch [15] and Thayer and Lane [16], those interactions cohere through nonlinear interdependencies to form a complex system comprising cortical, subcortical, and cardiac autonomic regulations [17,18]. As a matter of fact, the nonlinear dynamics in HRV have been shown to be a reliable substrate to explore how a particular architecture takes place across a largely distributed cognitive-autonomic network [19,20,21,22,23]. To date, researchers have attempted to grasp two main aspects of behavior in brain–heart interplay, namely, system coordination across multiple scales and a rich flux of information. So far, this information is supposed to lie in the HRV properties evaluated by fractal and entropy metrics, respectively [21,22,23,24,25,26]. Meantime, developing tools to reach a finer analysis through these metrics has been an active part of research in the last decades.

Since the introduction of detrended fluctuation analysis as a reliable method to capture scaling exponents that characterize fractal-like temporal structures in HRV [27], it has been recognized that complex phenomena in brain–heart interplay should not be oversimplified [28]. A multifractal behavior in heart rate variability [29] has been a common and interesting observation that deserves further scale-dependent analyses to take into account the crossover phenomena generally observed in short-range variations [28]. Scaling exponents in HRV may vary with the size of the observational scale, which led Castiglioni et al. to argue that a multiscale multifractal approach of HRV may provide a finer analysis of complexity in heartbeat dynamics [26,28]. By depicting a bidimensional multiscale and multifractal behavior in HRV, the mechanisms through which brain–heart interactions cohere during selective attention might be modeled more completely. In fact, multiscale multifractality was used recently to discriminate the conditions in which larger cascades of neural processes dominate in brain–heart interactions [23]. Through the analysis of the irregular behavior in HRV by using entropy metrics, these authors also inferred the robustness in information flux through the central autonomic network (CAN) neurovisceral integration. Computations of entropy adapted to relatively short RR time series [30] have been suggested to indicate how a cognitive task exacerbates RR entropy, a phenomenon that is abolished by a rise in anxiety [21,22]. To the best of our knowledge, the link between the possible benefits of a single session of SMR-NFb and neurovisceral integration during a task requiring selective attention has not been demonstrated.

Here, we aimed to show that improved attention, as a result of a single session of SMR-NFb, is reliably reflected in the multiscale multifractality of HRV dynamics (RR time series) because of the particular structuring of brain–heart interplay resulting from SMR-NFb. To illustrate this intuition with sufficient consistency, the efficacy of a single session of NFb was quantified. A rigorous use of the Stroop Color Word Test (SCWT) allowed the quantification of selective attention. A control (*Ctrl*) group was compared to the neurofeedback (*NFb*) (SMR-NFb training) group. Multiscale multifractality [26] and refined composite multiscale sample entropy [30] were computed from the HRV dynamics collected during the SCWT.

## 2. Materials and Methods

### 2.1. Participants

Thirty-five healthy students in sports sciences (20 females, 15 males, aged 21 ± 3 years old) gave their informed consent to take part in the present study that was part of their academic curriculum and for which they received credits. The IRB of the faculte des STAPS endorsed all the procedures, which followed the rules of the Declaration of Helsinki and its later amendments. All participants reported having no history of neurological or physiological disorders and normal or corrected-to-normal vision. None of them had ever experienced NFb training. The participants were asked to avoid alcohol and caffeine and to abstain from heavy physical activity for the 24 h preceding the experiment. The participants were randomly distributed in two groups. The *NFb* group (n = 17, age 21 ± 1 years, 10 females) underwent a single session of NFb for 20 min (as described below). Instead, the *Ctrl* group (n = 18, age 21 ± 4 years, 10 females) watched a neutral movie during this time.

### 2.2. Procedure

**Figure 1:**
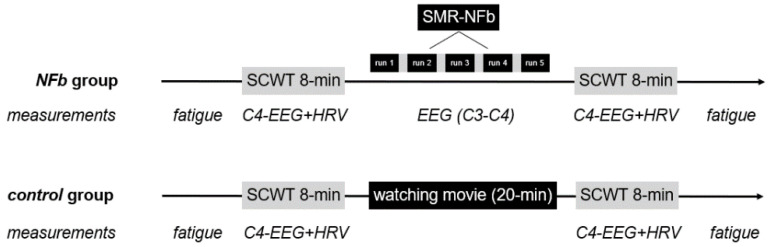
Procedure and measurements. SCWT, Stroop Color Word Test; SMR-NFb, neurofeedback training based on SMR frequency band. Fatigue was evaluated by an analog visual scale. HRV, Heart rate variability as collected by RR cardiac interbeat time series; EEG, electroencephalographic activities.

Before and after the intervention (NFb vs. watching a movie) the participants performed a Stroop Color and Word Test (SCWT) for 8 min while their heart rate variability was collected. For that, they were quietly seated on a chair at a viewing distance of about 0.5 m from the 15.6-inch screen of a laptop whose keyboard was used to respond to the stimuli of the SCWT displayed on the screen. To allow familiarization with the task, participants performed an initial SCWT for 8 min, guided by the experimenter to perfectly understand the task, mainly the instruction to respond as accurately as possible. Then, each participant performed two trials of SCWT, lasting 8 min, one before and one after the intervention: a single 20 min session of SMR-NFb in the *NFb* group and watching an emotionally neutral movie for the *Ctrl* group (Figure 1).

The overall procedure lasted about 40 min. In order to discard a potential confounding factor associated with mental fatigue, the participants were asked to self-report their feelings about fatigue at the beginning and at the end of the procedure (Figure 1).

### 2.3. Stroop Color and Word Test (SCWT)

The SCWT can be used to explore different cognitive functions depending on how the experimenter designs the test. Selective attention was the focus of the present study, which involved the subject selecting which information to process and to ignore during SCWT. The “selective attention theory” argues that color recognition requires more attention than word reading. Therefore, in our conditions, the participants were instructed to pay attention to correctly name the color “ink” of a word that designated a color, with the ’ink’ and the word sometimes designating the same color (a so-called congruent trial) or different colors (incongruent). A given ratio between incongruent and congruent response times has been proposed as a cue to infer that selective attention has been a dominant process during the task (see Section 3.2).

**Figure 2:**
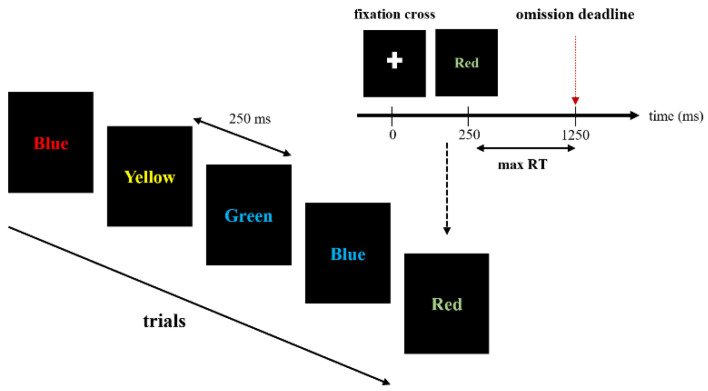
A typical sequence of the Stroop Color and Word Test (SCWT). max RT = maximum response time tolerated, after which the response was considered incorrect.

SCWT was implemented using the software PsyToolkit [31,32]. All stimuli were presented in the center of the screen displaying a black background color. Each trial began with the presentation of a fixation cross that was replaced by the word stimulus after 250 ms (Figure 2). During the task, a word naming a color among “blue”, “green”, “red”, or “yellow” appeared in an unpredictable manner, displayed either in a congruent (similar) color or in incongruent (different) color (blue, green, red, or yellow). The participants had to type on the keyboard where stickers indicated the corresponding colors before the displayed word disappeared (1000 ms) (Figure 2). The next word appeared on the screen 250 ms later.

SCWT performances were reported as two distinct dimensions: correct responses and response times. Both indices were computed considering all responses indistinctively and considering congruent and incongruent trials separately.

### 2.4. The Single Neurofeedback Session

#### 2.4.1. Description of SMR Training

**Figure 3:**
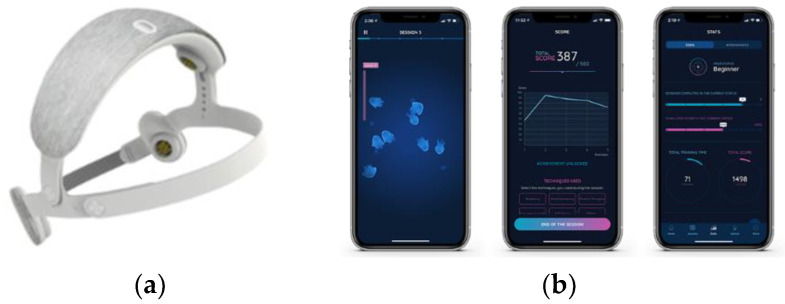
The URGOnight tele-neurofeedback device. The EEG headband (**a**) permits the recording of sensory-motor cortex electrical activity through four dry electrodes. Two of them are on the C3 and C4 positions, and the others are for reference (ground) and are placed on the mastoids. The mobile application (**b**) displays and emits visual and audio feedback according to the EEG fluctuations, and the app guides the neurofeedback training.

The single session of NFb lasted 20 min, during which five 3 min runs were used to guide SMR magnification, interspersed by 1 min rest periods. The participant was wearing a headband (Figure 3) (URGOnight, Urgotech, Paris, France) equipped with two electrodes located on the C3 and C4 positions and two electrodes located on the mastoids [33]. To reach SMR magnification, no specific instructions were given except to relax [34,35]. The participant had to focus on a vertical bar displayed on a smartphone, thanks to the dedicated app, coupled to auditory feedback that sent rewards each time the SMR power increased for 400 µs. The participants reached a higher level in the app (as in video games) each time the SMR power was maintained for 2 s. Conversely, they were visually informed of the absence of SMR magnification for 7 s, which brought them back to the lower level. This kind of gamification generally promotes rapid progress, which was carefully quantified here to evaluate NFb training efficacy.

#### 2.4.2. Index of NFb Training Efficacy

**Figure 4:**
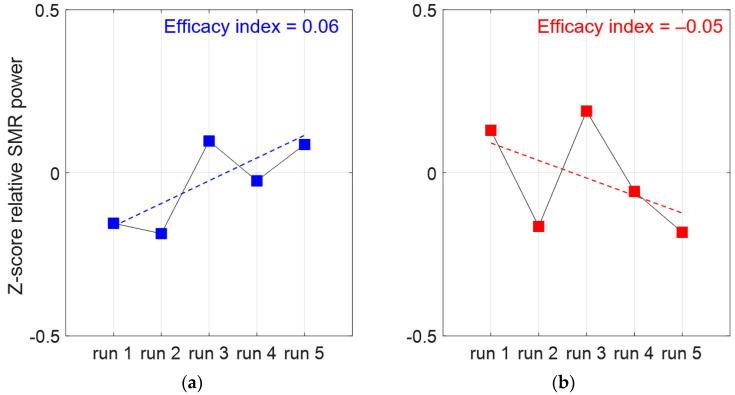
Two examples of individual efficacy quantification where linear fits of SMR relative power as a function of the five successive NFb runs show better efficacy in participant (**a**) when compared to participant (**b**).

As there is was risk that participants failed to achieve significant SMR magnification during training, an assessment of NFb efficacy was needed. Following the guidelines from Ros et al. [36], the within-session dynamics, meaning the dynamics of SMR production over successive runs, were quantified. For that, we used the method proposed by Reichert et al. [35] that consists of calculating the slope of a linear fit when a relative change in SMR power is represented as a function of the five successive NFb runs (Figure 4). Reichert et al. suggest that a better efficacy can be deduced from a higher slope, which makes sense with our “gamification” design during the guided NFb training (see Section 2.4.1). Therefore, the efficacy index corresponded here to the coefficient of a linear fit (slope), computed as follows:

First, for each run, the relative SMR power was computed as SMRpower (8–12 Hz)/Totalpower (8–40 Hz). Second, the above ratio was averaged among the collected C3 and C4 signals and calculated for each of the five runs (within session); then, these averaged ratios were expressed as a function of the session mean (Z-score, Figure 4). The NFb efficacy corresponds to the slope of the linear approximation computed over the five-run Z-score values, as shown in Figure 4. The greater the slope, the better the efficacy.

### 2.5. Frequency Domain Analysis of C4-EEG Time Series during SCWT

C4-EEG data were collected with an URGOnight headband at 200 Hz during the SCWT blocks for the NFb and Ctrl groups. This portable device was preferred to a conventional EEG device for practical considerations. Preprocessed C4-EEG time series were analyzed in the frequency domain by using the ensemble empirical mode decomposition (EEMD) method adapted from Huang [37]. This method discriminates a nonlinear and non-stationary signal into multiple intrinsic mode functions (IMF), allowing frequential components of the signal to be defined. By adding white noise to the signal to assist the decomposition (in the present study, an added white noise presenting a 0.1 standard deviation for 10 realizations and 200 maximum iterations), the EEMD method solves the mode mixing problem potentially described when using the EMD method initially described by Huang [37]. A detailed description of the EEMD procedure to analyze EEG signals can be found in [38] by Xiao-jun et al. After the decomposition of the C4-EEG signal into IMF, a Hilbert–Huang transform was used to compute the instantaneous frequency and energy of each IMF for each participant (*NFb* and *Ctrl* group) during each SCWT (SCWT-pre and SCWT-post).

### 2.6. HRV: Analysis of RR Time Series

Cardiac interbeat time intervals (RR) were collected during each SCWT in both the *NFb* and *Ctrl* groups (Figure 1) using a bipolar electrode transmitter belt Polar H10© (Polar^®^, Finland). The accuracy of this device has been demonstrated elsewhere [39]. The RR time series were imported to Matlab (Matlab Release 2021a, Mathworks, Natick, MA, USA) for subsequent analyses using existing Matlab functions and custom-designed algorithms. First, occasional ectopic beats (the irregularity of the heart rhythm involving extra or skipped heartbeats, e.g., extrasystoles and consecutive compensatory pause), were visually identified and manually replaced with interpolated adjacent values.

#### 2.6.1. Time and Frequency Domain Analyses of RR Time Series

The root mean square of the successive difference (RMSSD) in the RR time series that relies on the magnitude of the short-range fluctuations of heart rate associated with parasympathetic (vagal) modulation was computed. A discrete Fourier transform was computed after cubic spline interpolation to obtain a 4 Hz resampled RR time series. The power in the low-frequency (LF, [0.04–0.15] Hz) and high-frequency bands (HF, [0.15–0.40] Hz) were extracted. The power ratio LF/HF was calculated as an index of the sympathovagal balance [40].

#### 2.6.2. Entropy in RR Time Series and Their Shuffled Surrogates

The irregularity of the RR time series was evaluated using sample entropy computed over a range of observational scales [41] in experimental time series as well as in their shuffled surrogates. The series were first pre-processed given that pronounced non-stationarity could bias entropy measurement [42,43,44]. The drift in each series was subtracted as the residual of an empirical mode decomposition (EMD). After removing the drift, the signal stationarity was checked using the RWS method [42].

Then, the entropy in the pre-processed series was quantified using the refined composite multiscale entropy (RCMSE) method [30]. This method is better suited for the analysis of short time series, achieves better accuracy in entropy computations, and presents a lower risk of undefined entropy [30]. RCMSE was calculated as follows:

(1) $$RCMSE\left(x,\tau ,m,r\right)=-\ln\left(\frac{\sum_{k=1}^{\tau }n_{k,\tau }^{m+1}}{\sum_{k=1}^{\tau }n_{k,\tau }^{m}}\right),$$

where x is the RR time series, τ the scale factor, m the embedded dimension (here, m=2), and r is the tolerance factor (here, 0.15) [45]. The RCMSE value was calculated for each scale, τ, from τ=1 to τ=4. Since the length of the signal is critical for reliable entropy determination, the length of each analyzed time series was determined based on the shortest series among an individual set of recordings. Finally, we calculated an entropy index (Ei) from the area under the curve of RCMSE vs. scales, obtained by using the trapezoidal rule [22,23]. The same procedure was applied to 50 shuffled surrogates (mimicking white noise) of each RR time series to ensure that the RCMSE in the original series departed away from the RCMSE in the surrogates.

#### 2.6.3. Multiscale Multifractality in RR Time Series and Their Phase-Randomized Surrogates

The multifractal multiscale structure of the 1 Hz resampled [23] RR time series were analyzed by the fast DFA algorithm developed by Castiglioni et al. [26]. Given the resampled RR time series xi of length N  samples, we calculated its cumulative sum, yi. We split yi into M  maximally overlapped blocks of τ  seconds. We detrended each block with a least-square linear regression and calculated the variance of the residuals in each  k-th block, $\sigma^{2}\tau \left(k\right)$. The variability function, Fq(τ), is the q-th moment of $\sigma^{2}\tau$ [46]:

(2) $$Fq\left(\tau \right)={\left(\frac{1}{M}\sum_{k=1}^{M}{\left(\sigma_{\tau }^{2}\left(k\right)\right)}^{\frac{q}{2}}\right)}^{\frac{1}{q}}\;for\;q\;\neq 0,$$

(3) $$Fq\left(\tau \right)=e^{\frac{1}{2M}\sum_{k=1}^{M}\ln\left(\sigma_{\tau }^{2}\left(k\right)\right)}\;for\;q=0,$$

We evaluated Equation (2) for q between [−5; 5] and block sizes, τ, between 10 s and N/4  s. We evaluated the multifractal multiscale coefficients as a function of the time-scale, τ, α(q,τ), calculating the derivative of log Fq(τ) vs. log n [26].

The parameter q in Equations (2) and (3) defines the moment order calculated for the variances of the residuals. In the traditional monofractal DFA [27], the variability function corresponds to the second-order moment (i.e., q=2). While monofractal series exhibit the same α coefficient for all moment orders, q, in multifractal series, positive moment orders, q, weigh more in the contribution of the fractal components with greater amplitudes, and negative moment orders, q, weigh more in the contribution of the fractal components with lower amplitudes.

To define a multifractal degree as a function of scale, τ, MFi(τ) was obtained by calculating the standard deviation of all α(q,τ). Then, individual MFi profiles were observed as a function of time scales, τ. To compare conditions and groups, we computed a unique multifractal index called MFI by calculating the area under the curve (trapezoid rule) obtained between specific scales. In reference to 22, here, we selected τ=10 s to τ=17 s [23]

, which is quite similar to the alpha short-term coefficient reported by [47], τ=8 s to τ=16 s, but takes into account the excessive deviations introduced by the method in alpha values at the shortest scales, typically τ<10 s [26,28].

The proportion of non-linear components in the multifractal time series was computed by generating 50 surrogate series using the iterative amplitude adjusted Fourier-transformed method(IAAFT, [48]) from the 1 Hz resampled HRV time series.

### 2.7. Statistical Analyses

Values are expressed as means and standard deviations (SD). Outliers were identified using the interquartile range method [49] and replaced by the mean value for the considered sample. The prerequisites for parametric tests were checked using the Shapiro–Wilk test (normality) and the Levene test (homogeneity of variance in samples). To test the NFb training effect on attention, RR dynamics, and EEG frequential features, a two-way repeated measures ANOVA in a 2 × 2 design (a group between factor (*Ctrl* and *NFb*) and a time within factor (pre- and post-training) was computed. Holm’s correction for multiple comparisons was used as a standard post hoc test to neutralize the potential increase in type I error.

In addition to the classical frequential approach, Bayesian tests (log(BF10)) were computed to extend insight and guide the interpretation of the significance (*p*-values) according to the likelihood of the alternative hypothesis versus the null hypothesis [50,51] (see Bouny et al. [23] for an interpretation scale of this parameter adapted from Jeffreys, [52]).

A multiple regression analysis using the backward elimination technique was used to predict SCWT performance. The backward elimination begins with all variables of the model. At each stage, the variables that contribute the least to the discriminatory power of the model are removed. If all the remaining variables meet the remaining criteria in the model, the process is stopped. In addition, we rigorously conformed to the basic assumptions of the regression model: linearity, independence, continuity, normality, homoscedasticity, autocorrelation, and outliers. The significance of the model was verified using the *F*-test for each multiple regression model developed. The coefficients of determination (*R*^2^), adjusted coefficients of determination (adjusted *R*^2^), and standard error of estimate (SEE) for the estimated multiple regression model were calculated.

Because in the presence of multicollinearity the solution of the regression model becomes unstable, the variance inflation factor (VIF) was computed. The VIF measures how much the variance of a regression coefficient is inflated due to multicollinearity.

The data were analyzed using JASP (version 0.16.2.0, https://jasp-stats.org, accessed on 12 May 2022) and R (version 4.2.0, R: The R Project for Statistical Computing (r-project.org) accessed on 12 May 2022).

## 3. Results

### 3.1. Index of NFb Efficacy

A significant positive slope in SMR power vs. training runs was observed in 60% of the participants, who therefore demonstrated a high capacity to improve the level of SMR generation as the runs were repeated. The heterogeneity observed in individual NFb efficacy provided the opportunity to test NFb efficacy in relation with gains in SCWT performance (Section 3.6).

### 3.2. Self-Reported Feeling of Fatigue

**Figure 5:**
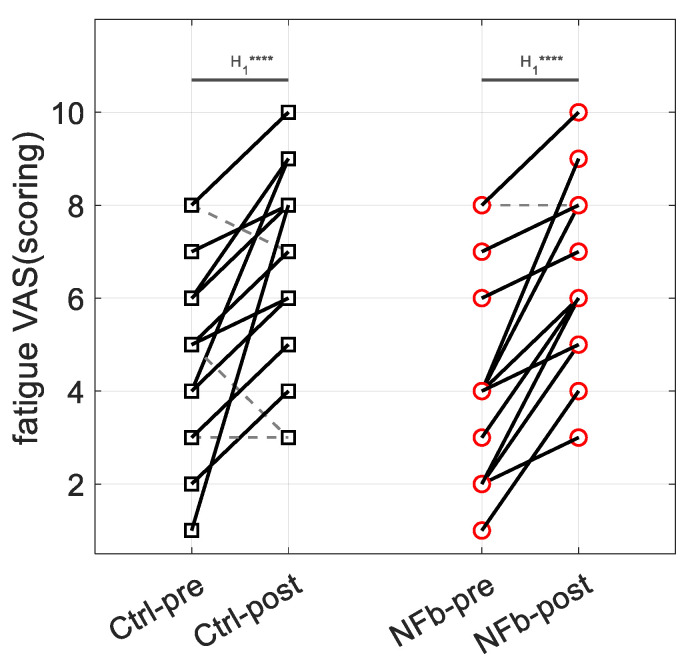
Effect of the intervention on subjective fatigue report by visual analog scale (VAS) of fatigue for the control (Ctrl) and the neurofeedback (NFb) groups. Dashed gray lines indicate decreases while solid black lines indicate increases. Interpretation scale: H1 **** means extreme evidence for the alternative hypothesis.

A two-way (pre vs. post x NFb vs. movie) ANOVA indicated increased levels of self-reported fatigue in each group at the end of the experimental procedure (Figure 5) (simple main effect of time within factor—F = 50.68, *p* < 0.001; log(BF10) = 12.76) and the absence of group-specific increase (interaction effect—F = 0.36, *p* = 0.55; log(BF10) = −0.59).

### 3.3. Performance in SCWT

We distinguished two dimensions in SCWT performance in both congruent and incongruent trials: the ratio of correct responses and respective response times.

#### 3.3.1. Correct Responses

**Figure 6:**
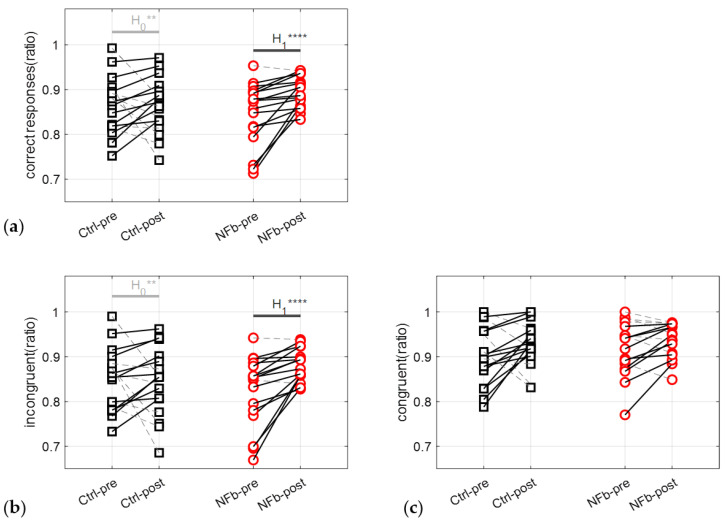
Proportion of correct responses provided by each individual participant in the pre- and post-training Stroop color and words tests (SCWT) for all trials (**a**), only incongruent trials (i.e., ink color does not match the displayed color word) (**b**) and only congruent trials (i.e., ink color matches the displayed color word) (**c**). For all trials (**a**) and incongruent trials (**b**), the *NFb* group showed significantly better performances than the control (*Ctrl*) group after training. For congruent trials, no differences were observed. An individual improvement in performance is highlighted by a solid line; a decline in performance is highlighted by a dashed gray line. Interpretation scale: In panels (**a**) and (**b**), H1 **** and H0 ** mean, respectively, extreme evidence for the alternative hypothesis and strong evidence for the null hypothesis. No evidence in panel (**c**).

The ratio of correct responses was significantly improved after NFb, with an interaction effect of group (*Ctrl*/*NFb*) x time (pre-/post-training) (F = 5.22, *p* < 0.05; log(BF10) = 0.39) (Figure 6a). The post hoc analysis indicates that subjects in the *NFb* group significantly improved their ratio after NFb training compared to the Ctrl group (*t* = −3.24, *p* < 0.05; log(BF10) = 3.33).

Going one step further by focusing on congruent and incongruent trials, it is worth noting that only the ratio of incongruent trials was improved after NFb, with an interaction effect of time x group (F = 6.77, *p* < 0.05; log(BF10) = 0.56) (Figure 6b)). Subjects in the *NFb* group significantly improved the ratio of incongruent responses after NFb training compared to the Ctrl group (*t* = −3.32, *p* < 0.05; log(BF10) = 3.48).

#### 3.3.2. Response Times

The two-way ANOVA (pre vs. post × NFb vs. movie) applied on SCWT response times indicated an absence of an NFb effect on these parameters. NFb training did not modify response times on SCWT among all trials or for the congruent or incongruent trials compared to the *Ctrl* condition (Table 1).

### 3.4. Frequencies and Energy on C4-EEG during SCWT

**Figure 7:**
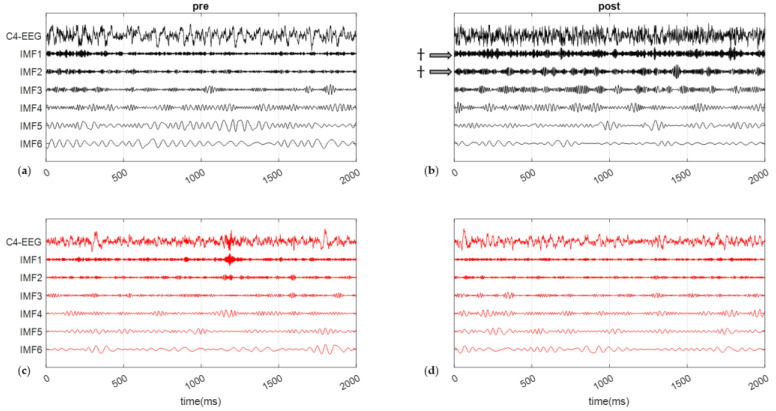
Empirical mode decomposition of typical C4-EEG signals (a 10 s window) into six intrinsic mode functions (IMF). Signal recorded before (**a**) and after (**b**) a control movie for a subject from the *Ctrl* group and before (**c**) and after (**d**) the SMR-neurofeedback session for a subject from the *NFb* group. Arrows and ^†^ indicate a significant evolution of the magnitude of the associated IMF. Here, the window size of M = 2000 corresponds to a 10 s epoch.

C4-EEG signals, recorded during the SCWT blocks thanks to the URGOnight, were decomposed into intrinsic mode functions (IMF, Figure 7). Both the IMF instantaneous frequency and instantaneous energy were calculated from the Hilbert–Huang transform for each SCWT (SCWT-pre and SCWT-post) (Table 2).

The instantaneous energies of IMF1 and IMF2 did not change after the session for the *NFb* group but were significantly decreased for subjects in the *Ctrl* group, with a significant time × group interaction (F = 10.70, *p* < 0.01; log(BF10) = 1.68 and *t* = −3.71, *p* < 0.01; log(BF10) = 1.75 for the IMF1, F = 9.66, *p* < 0.01; log(BF10) = 1.68 and t = −3.31, *p* < 0.05; log(BF10) = 1.84 for the IMF2). As illustrated in Table 2, subjects in the *NFb* group better maintained C4-EEG activity in the highest frequencies (50 Hz and 30 Hz) compared to *Ctrl*. These bands correspond, respectively, to gamma and beta cerebral waves, which are associated with information processing (gamma) and attention processes (beta) in cognitive tasks.

No differences were found for the other IMF frequencies.

The capacity of subjects in the *NFb* group to maintain cerebral activities in both IMF1 and IMF2 after NFb was not correlated with the index of NFb efficacy or, consequently, to the subject’s ability to produce SMR.

### 3.5. Heart Rate Dynamics during SCWT

#### 3.5.1. HRV Analysis in Time-Domain and Frequency-Domain

Although vagal and sympathetic markers changed in post vs. pre conditions in each group, a two-way (pre vs. post × NFb vs. movie) analysis of variance showed that HRV markers in time and in frequency domains failed to show any specific effects of NFb (Table 3). Overall, HF power and LF power (thus total power in HRV) tended to increase after both watching a movie and NFb session, with no change in LF/HF (sympathovagal balance), but this happened indistinctively in the *Ctrl* and the *NFb* group.

#### 3.5.2. Entropy in HRV

Complexity was quantified by entropy (RCMSE, marker: Ei) and the self-similarity multifractal multiscale DFA (marker: MFI) during both SCWT-pre and SCWT-post.

The entropy marker (Ei) did not differ between groups or experimental conditions. NFb training did not modify entropy markers compared to watching a movie.

The Bayesian factor confirmed this result by supporting strong evidence for the null hypothesis for the main time effect on the RCMSE measure (log(BF10) = −1.34) and very strong evidence for the interaction effect (log(BF10) = −3.21).

#### 3.5.3. Scale-Specific Multifractality in HRV

The multiscale multifractal marker MFI, obtained from HRV during SCWT, significantly decreased in the *Ctrl* group but reached similar values before and after the NFb session (*t* = −0.26, *p* = 1.00; log(BF10) = −1.08), with a significant interaction effect of time x group (F = 6.59, *p* < 0.05; log(BF10) = 0.99), showing a loss of multifractality in the *Ctrl* group (*t* = 3.42, *p* < 0.01; log(BF10) = 2.23). In order to depart from background noise as a possible explanation of this effect, we performed the same ANOVA on the average values of 50 phase-randomized (IAAFT) surrogates of each original series. After phase-randomization, as expected, the ANOVA showed that the NFb effect disappeared (F = 0.05, *p* = 0.83; log(BF10) = 0.14). Therefore, the NFb effect observed in the MFI of the original series was not fortuitous.

**Figure 8:**
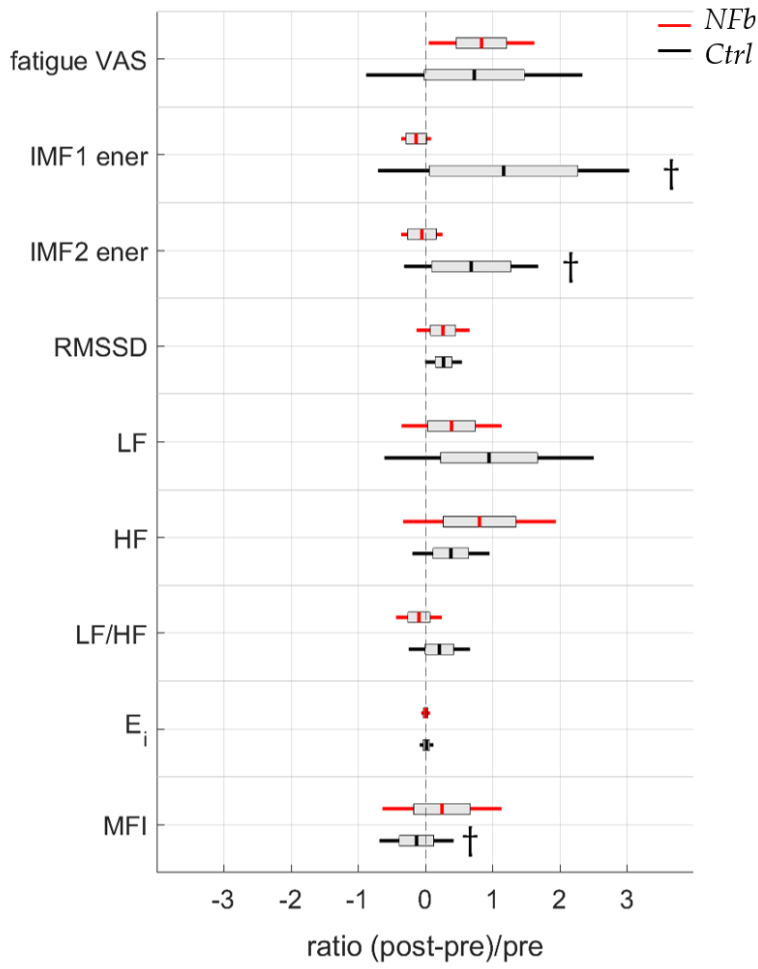
Psychological (visual analogic scale (VAS) of fatigue) and neurophysiological (from C4-EEG and HRV) feature evolution from before and after a neurofeedback training for the *NFb* group or a control movie for the *Ctrl* group. IMF = intrinsic mode function; RMSSD = root mean square of successive differences; LF = low-frequency power in HRV power spectrum; HF = high-frequency power in HRV power spectrum; LF/HF = ratio of low frequencies and high frequencies; Ei = entropy index; MFI = multifractal index. ^†^ indicates a significant difference considering interaction between time within factor and group between factor.

The relative evolution of the neurophysiological (EEG and HRV) and psychological (fatigue) parameters that could explain the observed changes in cognitive performance are illustrated in Figure 8.

These parameters have been integrated in a multiple linear regression model using backward elimination to define the best predictors of attention performance enhancement. This methodology is presented in the section below.

### 3.6. Multiple Linear Regression

Multivariate linear regressions using backward data entry were performed, considering the *NFb* group, to determine potential predictors of NFb-induced changes in SCWT performance. When considering the ratio of correct responses among all trials, changes in multifractality (∆MFI = MFI_pre_ − MFI_post_) was the stronger predictor, in association with individual ability to generate SMR during NFb training, quantified here as “efficacy” (Equation (4), F = 10.49, *p* < 0.05).

As a major outcome, it is worth noting that ∆MFI on its own was a strong predictor of performance when focusing on the ratio of correct answers on incongruent trials (Equation (5) F = 18.663, *p* = 0 < 0.01).

∆ NFb-induced changes in SCWT_all trials_ = 0.039 + (0.062 × ∆MFI) + (0.117 × efficacy) (4)

∆ NFb-induced changes in SCWT_Incongruents_ = 0.064 + (0.176 × ∆MFI) (5)

Although we noted a weak collinearity between ∆MFI and efficacy, which was too low to affect the validity of the model in Equation (4) (VIF = 1.96), we thought it was interesting to note that a positive correlation existed between training efficacy and ∆MFI (r = 0.70, *p* < 0.05). The more a participant was able to produce SMR during NFb, the more he maintained heartbeat complexity, and, based on Equation (5), SCWT performances, thus highlighting selective attention.

## 4. Discussion

As a main finding in the present study, we show that a single session of neurofeedback training (NFb) improved selective attention, which was tightly coupled with the emergence of a new dynamic structuring of brain–heart interplay. The links we observed between NFb efficacy, an NFb-induced gain in cognitive performances during SCWT, and the multifractal behavior of HRV add significant value to conclude the real effects of a single SMR-NFb session in our conditions, beyond a placebo effect, and go beyond a simple observation to suggest a mechanistic neurovisceral support. Although studies exploring a single session of SMR-NFb are scarce to date, our results extending the data to heart–brain interplay are coherent with a recent initiative extending self-reported and EEG improvements to cortisol regulation [11]. Both studies report on EEG correlates and go beyond these local effects to argue for the remodeling of much larger neurovisceral networks after a single session of NFb.

SMR-NFb training is a brainwave technique whose main effects on neural processes have been associated with the attenuation of somatosensory information flux to the cortex during SMR activity [3,8,9,11], which can persist in the form of cognitive and behavioral improvements after the training session [54]. The underlying mechanisms supporting a persistent effect are still poorly described to date regarding a single NFb session. Most of the time, mechanisms must be inferred from experimental conditions, the dispositions of participants (e.g., the healthy or diseased), and EEG correlates, but inconsistent results can make it difficult to organize insights in a coherent way. Goncalves et al. [55] observed no effect in healthy participants completing the attention network task. By contrast, a recent study showed a feeling of calm and reduced tension that was coherently associated with persistent EEG enhancements in the SMR band and reduced levels of salivary cortisol [11]. Less cortisol accumulation in the organism, a process modulated by the hypothalamic–pituitary–adrenal axis, indicates that the positive effects of a single NFb session are not confined to a few areas of the brain but rather rely on processes that unfold across a neurovisceral network where the adrenal glands represent the end effector. Our approach also built on NFb consequences across a neurovisceral integration, using the heart as an end effector, and focused on HRV as a source of information. Although part of the information may be grasped by the classical mode decomposition of HRV, the way dynamic interactions cohere through an intricate cognitive-autonomic network has been better described by fractal and entropy metrics in recent studies [21,22,23,24,25,26]. In line with this intuition, here, we found no relevant information in the time and frequency domains of HRV in response to NFb but rather entropy and fractal metrics in HRV helped elaborate on the integrated effects of NFb. The reasoning is deeply rooted in recent links established between cognitive activity, autonomic control, and HRV [21,22,23]. The level of irregularity in HRV is classically assessed by entropy metrics [41]. This level is thought to describe the activity of the CAN and its interference with cognitive modulations [19,22]. A situation where anxiety-driven changes in amygdala activity interfere with cognitively driven processes leads to a significant reduction in HRV irregularity [21]. Since in our conditions entropy did not change after NFb, the reduction in anxiety might not be the main factor restructuring the main network supporting a sharpened attention during SCWT (Figure 6). There is no discrepancy with the recent observation by Gadea et al. [11] that a reduction in anxiety can result from a single NFb session because our participants were cognitively active during the experimental measurements, though they were evaluated in resting conditions in the later study. Here, when facing SCWT requirements, anxiety reduction may not represent a dominant effect participating in the restructuring of the neurovisceral system, which is associated with better selective attention. Rather, by showing substantial changes in the scale-specific multifractality of HRV, other mechanisms are suspected. With a multifractality during SCWT that was better maintained in participants of the *NFb* group, the positive effects of a single NFb session most likely concern the ability to preserve critical interconnections in structuring the emergence of selective attention. By considering the degree of multifractality at distinct time scales of HRV, Castiglioni et al. [26,28] claimed to have developed a fine way to analyze multifractality in heart rate dynamics. Particularly in the short-range of HRV where scale-free and mode-locked processes are intertwined, evaluating scale-specific multifractal behavior has been valued when it comes to depicting a cognitive-autonomic architecture [23]. In agreement with Bouny et al. [23], the restructuring of this network seemed to operate across scales 10-to-17 s after NFb, as indicated by levels of multifractality (Table 3). The ability of participants in the *NFb* group to maintain normal levels during the post-SWCT while said level dropped in the *Ctrl* group appeared as the main correlate of a gain in selective attention (Equation (5)). When taking account of individual behavior during the single NFb training session, these two combined variables were also strong predictors of SCWT gains (Equation (4)). The multifractal properties in complex system behavior have been linked to adaptability [56]. It should be concluded that NFb in our conditions allowed the maintenance of a flexible cognitive adaptation to SCWT, where this particular ability was dropped in participants of the *Ctrl* group who lost part of their adaptability during the post-SCWT. The loss of adaptability in the *Ctrl* group may be explained by fatigue, a feeling that was self-reported in each group (Figure 5). By maintaining the complex structuring of neurovisceral integration in the NFb-trained participants who declared a similar level of fatigue, NFb might facilitate the emergence of cognitive functions [23], including selective attention. A specific network has been identified by neuroimaging studies and called the sustained attention network (SAN), including cortical areas (prefrontal cortex, anterior insula, and parietal areas) and subcortical structures (cerebellar vermis, thalamus, putamen, and midbrain) [13]. By using neuroimaging-based feedback to selectively activate this core network while concomitantly deactivating the default mode network (mind-wandering), an alternative method to the presently used SMR-NFb showed that it is possible to improve sustained attention [57]. Since SMR-NFb facilitates the modulation of thalamocortical networks [3], it might be proposed that this NFb technique improves the brain–heart interplay specifically in the selective attention parts of the SAN.

The C4-EEG analysis during SCWT provides additional evidence about the role of NFb in counteracting the onset of mental fatigue. In particular, high-frequency modulations are affected by mental fatigue [58], and these particular frequencies were better maintained during the 8 min SCWT in the *NFb* group (Table 2). To resume in our conditions, while fatigue deteriorated the neural capacities of activation as well as neurovisceral networked coordination, a single session of NFb practice helped counteract these effects to the benefit of a neurovisceral structuring in favor of the emergence of selective attention.

As a whole, the present study also helped to assess the single-session NFb technique beyond any placebo effect and may help structure future NFb interventions. Despite obvious interest in NFb training for health and well-being, the practice in itself may appear unattractive as long as a significant number of daily repeated sessions is needed. The individual ability to generate SMR waves with sufficient propensity during training is another drawback. Our results might pave the way for one-shot NFb practice, inviting a rigorous control on training efficacy. Thanks to the visual NFb training provided by the headset/smartphone device, here, 60% of the NFb participants were able to generate more and more SMR during five short successive trials in a single session (Figure 3), though they had no previous experience with NFb. This means that NFb practicability can be strengthened over years thanks to the development of user-friendly connected devices.

## 5. Conclusions

Using the SCWT as an experimental challenge, our study elaborated on cognitive-autonomic interactions to grasp the NFb effects on the cognitive dispositions of young healthy subjects. Our observations add to recent intuitions that a single SMR-NFb session improves brain modulations, as reflected in both self-reported feelings and neurophysiological correlates. It is speculated that the underlying mechanisms may involve two critical networks known as SAN and CAN with common structures that are jointly stimulated by SMR-NFb, which may provide a neurophysiological background explaining the improvement in selective attention.

However, this study is not without limitations; since we used a home-based device designed for non-clinical use, using a sham-controlled group, as consensually recommended by guidance, was not possible. Rather, we used a control group watching a cognitively and emotionally neutral documentary. Better evidence of the NFb effect could also be obtained by replicating this experiment with a broader sample of participants, which would strengthen the observed effect size.

To conclude, adding to a recent work showing improved mood modulations using a single session of SMR-NFb to achieve anxiety reduction, we suggest here that other mechanisms might operate through a new structuring of heart–brain interplay that supports the emergence of better selective attention. Further studies are needed to explore which neurophysiological processes can be remodeled after a single session of NFb. They might be inspired by the fact that improved selective attention is useful on its own and in combination with other cognitive processes, such as those involved in learning.

## Figures and Tables

**Table 1:** Descriptive statistics (means ± standard deviations) of SCWT response times.

SCWT Response Times (Unit)	Ctrl (N=18)		NFb (N=17)	
	Pre	Post	Pre	Post
RTtotal (ms)	616 ± 55	606 ± 64	640 ± 34	611 ± 36
RTcorrect (ms)	614 ± 54	604 ± 63	633 ± 33	606 ± 35
RTerror (ms)	664 ± 139	656 ± 99	703 ± 40	669 ± 56
RTcongruent (ms)	584 ± 51	571 ± 53	597 ± 42	575 ± 38
RTincongruent (ms)	629 ± 60	621 ± 70	652 ± 65	623 ± 34

RTtotal = average response time among all trials; RTcorrect = average response time for correct responses only; RTerror = average response time for wrong responses only; RTcongruent = average response time for congruent trials (i.e., ink color matches the displayed color word); RTincongruent = average response time for incongruent trials (i.e., ink color does not match the displayed color word); SCWT = Stroop color and word test.

**Table 2:** Means (±standard deviations) of frequency and energy values of the six IMFs computed based on the ensemble empirical mode decomposition method [53] conducted on the cerebral activity in the sensory-motor cortex (C4). IMFs were calculated for each group of subjects on pre- and post-NFb training or watching a movie.

IMFs	Ctrl (N=18)				NFb (N=17)			
	Frequency (Hz)		Energy		Frequency (Hz)		Energy	
	Pre	Post	Pre	Post	Pre	Post	Pre	Post
IMF1	50.7 ± 1.6	51.3 ± 2.6	21.8 ± 17.2	42.5 ± 35.6 ^†^	51.0 ± 1.6	52.0 ± 1.2	44.5 ± 23.2	36.9 ± 18.8
IMF2	30.0 ± 1.1	30.6 ± 1.8	18.3 ± 10.5	27.9 ± 16.2 ^†^	31.4 ± 1.5	31.6 ± 1.0	23.8 ± 13.8	20.0 ± 6.8
IMF3	17.7 ± 0.4	18.2 ± 1.2	17.2 ± 6.7	21.7 ± 8.4	18.4 ± 0.9	18.8 ± 1.2	19.8 ± 7.0	17.7 ± 5.8
IMF4	10.3 ± 0.6	10.6 ± 0.9	18.6 ± 5.0	20.0 ± 5.3	10.8 ± 0.6	10.8 ± 0.4	18.5 ± 3.1	18.1 ± 5.7
IMF5	5.9 ± 0.4	6.2 ± 0.5	19.7 ± 5.5	18.5 ± 3.4	6.2 ± 0.5	6.3 ± 0.3	23.3 ± 9.3	18.7 ± 4.7
IMF6	3.5 ± 0.2	3.6 ± 0.2	16.5 ± 4.6	14.8 ± 3.4	3.6 ± 0.2	3.8 ± 0.3	19.2 ± 3.5	19.5 ± 4.1

IMF = intrinsic mode function. ^†^ indicates a significant difference considering the interaction between time within factor and group between factor.

**Table 3:** Mean (±standard deviation) of time-(RMSSD) and frequency (Low Frequencies: LF, High Frequencies: HF, sympatho-vagal ratio: LF/HF) domains indices and nonlinear complexity indices (entropy index, Ei and multifractality, MFI) computed from RR time series.

HRV Variables (Unit)	Ctrl (N=18)		NFb (N=17)	
	**Pre**	**Post**	**Pre**	**Post**
RMSSD (ms)	51.4 ± 24.0	62.8 ± 27.2 *	47.2 ± 20.2	54.7 ± 17.0 *
LF (ms^2^Hz)	1399 ± 764	2374 ± 1660 *	1618 ± 1103	1943 ± 1351
HF (ms^2^/Hz)	1041 ± 795	1339 ± 947 *	727 ± 486	1097 ± 711 *
LF/HF	1.50 ± 0.62	1.68 ± 0.72	2.28 ± 0.85	1.97 ± 0.85
Ei (u.a.)	5.98 ± 0.62	6.02 ± 0.21	6.11 ± 0.31	6.13 ± 0.47
MFI (u.a.)	0.65 ± 0.40	0.42 ± 0.17 ^†^	0.48 ± 0.23	0.50 ± 0.19

RMSSD = root mean square of successive difference; LF = low frequencies power in HRV power spectrum; HF = high frequencies power in HRV power spectrum; LF/HF = ratio of low frequencies on high frequencies; Ei = entropy index; MFI = multifractal index. * indicates a significant difference considering time within factor, ^†^ indicates a significant difference considering interaction between time within factor and group between factor.

## Data Availability

The datasets generated and analyzed during the current study are available from the corresponding author on reasonable requests.
